# A Novel Mice Model for Studying the Efficacy and IRAEs of Anti-CTLA4 Targeted Immunotherapy

**DOI:** 10.3389/fonc.2021.692403

**Published:** 2021-06-10

**Authors:** Shengchao Xu, Xi Yan, Gan Dai, Chengke Luo

**Affiliations:** ^1^ Department of Neurosurgery, Xiangya Hospital of Central South University, Changsha, China; ^2^ Health Management Center, Xiangya Hospital of Central South University, Changsha, China; ^3^ Department of Microbiology, Xiangya School of Medicine, Central South University, Changsha, China

**Keywords:** glioblastoma, humanized patient-derived orthotopic xenograft, immune-related adverse events, anti-CTLA4 immunotherapy, regulatory T cells

## Abstract

**Background:**

Patient-derived orthotopic xenograft (PDOX) is a popular animal model for translational cancer research. Immunotherapy is a promising therapy against glioblastoma (GBM). However, the PDOX model is limited to evaluating immune-related events. Our study aims to establish GBM humanized PDOX (HPDOX) mice models to study the mechanism of anti-CTLA4 immunotherapy and immune-related adverse events (IRAEs).

**Methods:**

HPDOX models were established by culturing GBM tissues and intracranially implanting them in NSG mice. Meanwhile, peripheral blood mononuclear cells (PBMCs) were separated from peripheral blood and of GBM patients and administrated in corresponding mice. The population of CD45+, CD3+, CD4+, CD8+, and regulatory T (Treg) cells was estimated in the peripheral blood or tumor.

**Results:**

T cells derived from GBM patients were detected in HPDOX mice models. The application of anti-CTLA4 antibodies (ipilimumab and tremelimumab) significantly inhibited the growth of GBM xenografts in mice. Moreover, residual patient T cells were detected in the tumor microenvironment and peripheral blood of HPDOX mice and were significantly elevated by ipilimumab and tremelimumab. Additionally, Treg cells were decreased in mice with IRAEs. Lastly, the proportion of CD4+/CD8+ T cells dramatically increased after the administration of ipilimumab. And the degree of IRAEs may be related to CD56+ expression in HPDOX.

**Conclusions:**

Our study established HPDOX mice models for investigating the mechanism and IRAEs of immunotherapies in GBM, which would offer a promising platform for evaluating the efficacy and IRAEs of novel therapies and exploring personalized therapeutic strategies.

## Background

Glioblastoma (GBM) is one of the most malignant primary brain cancers. The median overall survival of GBM patients is only 14.6 months, despite the satisfactory surgery and concomitant chemoradiotherapy (1–3). Therefore, there is a clear urgent to reveal the mechanism of tumorigenesis and development and develop novel therapeutic agents against GBM. However, recent clinical trials without appropriate mice models in this area have resulted in dissatisfactory and inconsistent therapeutic effects. It cannot reflect the tumor microenvironment, principal histologic, and genetic characteristics of GBM. In this context, the development of accurate and reproducible animal models is essential.

The patient-derived orthotopic xenograft (PDOX) has attracted more and more attention to facilitate biologic studies, preclinical drug evaluation, and biomarker identification (4–8). Nevertheless, clinically relevant PDOX models are not fully capable of recapitulating patients’ immune systems, which impedes the evaluation of immunotherapy efficacy. In recent years, although the co-cultures combined GBM organoids with immune cells such as human peripheral blood mononuclear cells (PBMCs) have been of great concern, which could be an ideal platform for immunotherapy selection (9), since the tumor-derived spheres are still not in the same conditions such as hypoxia or immune microenvironment, as to those occur in intracranial. Besides, the co-culture conditions combined GBM organoids with immune cells are not the optimum one for each cell type according to compromise strategy (10, 11). Meanwhile, immune-related adverse events (IRAEs) arising under immunotherapy forcing us potentially without precedent to think of strategies to maintain the immune system. Therefore, revealing the mechanism of T cells from PBMCs in the action of IRAEs and anti-tumor immunity is essential to balancing the immune system in developing a cancer immunotherapy strategy.

Humanized PDOX models (HPDOX) were regarded as the next-generation PDOX. Although humanized mice were reported in few tumors such as myeloma and hepatocellular carcinoma, humanized GBM mice models were seldom studied in recent literature (12). Those models were essential to study the potential and limitations for differential immune-enhancing approaches, as well as contribute to refining the framework of emerging immunotherapy strategies and related IRAEs against GBM. Our study was conducted to establish humanized GBM mice models and investigate the efficacy and IRAEs of anti-CTLA4 immunotherapy. Our studies aimed to provide a platform to develop effective strategies to minimize immune therapeutic IRAEs without impeding anti-tumor immunity in the future.

## Materials and Methods

### Human GBM Tissue Specimens

Fresh GBM specimens were obtained from patients who received surgery in the Department of Neurosurgery, Xiangya Hospital from 2016 to 2020. All clinical samples were collected with informed consent obtained from the patients. All procedures were conducted following the Declaration of Helsinki (1964).

### Peripheral Blood

All procedures were approved by the Ethics Committee of Xiangya Hospital. Peripheral blood (PB) specimens were collected from patients who received surgery in the Department of Neurosurgery, Xiangya Hospital from 2016 to 2020 with written informed consent obtained. PBMCs were isolated using Lymphoprep (Stem Cell Technologies) according to the manufacturer’s instructions.

### T Cell Reconstitution and Anti-CTLA4 Antibodies

The reconstitution of CD4+ and CD8+ T cells of PB from mice was monitored every week. A total of 1 × 10^6^ GBM patients’ PBMCs were implanted into sub-lethally irradiated (0.5 Gy) 4–6-week-old male NSG mice by tail vein. The anti-CTLA4 antibodies (ipilimumab, ipilimumab with N298A mutation, and tremelimumab) were generous gifts from Huntsman Cancer Institute, University of Utah, USA.

### Generation of HPDOX Mice Model

Some 4–6-week-old male NSG (NOD.Cg−*Prkdc* ^scid^ *Il2rg* ^tm1Wjll^/SzJ) mice were used in this study. All animal experiments were obtained and performed at the Laboratory Animal Center of the Central South University and all procedures were approved by the Ethics Committee of Xiangya Hospital following the Guide for the Care and Use of Laboratory Animals. PDOX was established from surgically resected specimens in NSG mice. Fresh surgical specimens were rinsed with Hank’s solution three times. Then the tumor tissue was cut into several 1–3 mm^3^ pieces. The tissue was incubated with accutase for 30 min at 37°C and dissociated into single cells. Cells were cultured in serum-free medium (Canada, Stemcell Technologies) in an incubator. Before transplantation, cells were digested and resuspended in the medium with a density of 1 × 10^8^/ml. The injection was located at the skull 1–2 mm lateral and 1 mm anterior to the bregma. Each time, 5 μl of cell suspension was aspirated using the Hamilton syringe and slowly injected into the brain at a dept of 2–3 mm with a rate of 1 μl/min. After the completion of the injection, maintain the needle for 3 min before withdrawing to reduce the backflow of the injected cells spillover. After 14 days of PDOX established, the mice with criterion can be incorporated for further study: (a) The weight loss is no more than 10%; (b) Motor function is normal, without hypokinesis; (c) Hair’s clean and shiny, without ruffings; (d) The bowel and bladder functions are normal.; and (e) No signs of infection or any other illness. To estimate the tumor volume, the simple random sampling method was employed to select three mice from each group for sacrifice. The tumor volume was calculated according to the HE slides with the greatest cross-sectional area. Tumor volume was determined using the following equation: length (L) × width^2^ (W) × 0.5. Tumor volume of the three mice was within our expectation, whereas some mice were excluded because the mice failed to meet the standard as described previously. There is no significant difference in tumor volume at this time point (**Figure S1**, P >0.05). Afterward, the HPDOX mice model was established by injecting PBMCs from GBM patients at 4 h after irradiation (13). The antibodies were given twice a week after PBMCs transplantation. The mice in our study were monitored for up to 3 months for weight, health, or immune status. HPDOX suffered systemic IRAEs were assessed by the criterion of mice graft-versus-host disease (GVHD) clinical scoring system including weight loss, posture, activity, fur texture, and skin integrity (14). The mouse’s total score was estimated twice a week. Mice were sacrificed according to the following criteria: weight loss of 20–25%; tumor weight reached 10% of mice weight; appetite loss of more than 24 h; depression and hypothermia. The observation endpoint was defined as 104 days (14 days for model establishment and 3 months for observation).

### Flow Cytometry

PB containing the anticoagulant sodium heparin was centrifuged at 1,500 rpm for 5 min at room temperature. Red blood cells were lysed by Red Blood Cell Lysis Buffer (Sigma), and the white blood cells were pelleted at 300*g* for 3 min. Flow cytometric analysis was performed using the LSR FORTESSA device (BD Biosciences, San Jose, CA, USA). The samples were incubated with the following antibodies to identify T and regulatory T (Treg) cells: anti-human CD3 FITC (BD Biosciences), anti-human CD4 Pacific Blue (BD Biosciences), anti-human CD8 APC (BD Biosciences), anti-human CD45 PE (BD Biosciences), IFNγ-APC (BioLegend), CD45RA- PE (BD Biosciences), CD45RO-APC-cy7 (BD Biosciences), anti-human FOXP3 PE (BD Biosciences), CFSE FITC (BD Biosciences), CD25 PE (BD Biosciences), CD127 PE-cy7 (BD Biosciences), CD56 PE (BD Biosciences), and HLA-DR PE-cy7 (BD Biosciences). The following controls were used: unstained cells and single-stained cells; and dead cells, which in conjunction with AutoComp software were used to set accurate compensation and data analysis. Cells were counted per sample, and the data were analyzed with FlowJo V10.

### Statistical Analyses

Statistical analyses were conducted using GraphPad Prism v8.0. One-way ANOVA or unpaired two-tailed Student’s t-test was used to estimate the difference between two or more groups. Kaplan–Meier analysis was used to evaluate the survival difference between two groups. Two-sided p <0.05 was considered as statistical significance.

## Results

### Establishment of GBM HPDOX Mice Models

**Figure 1:**
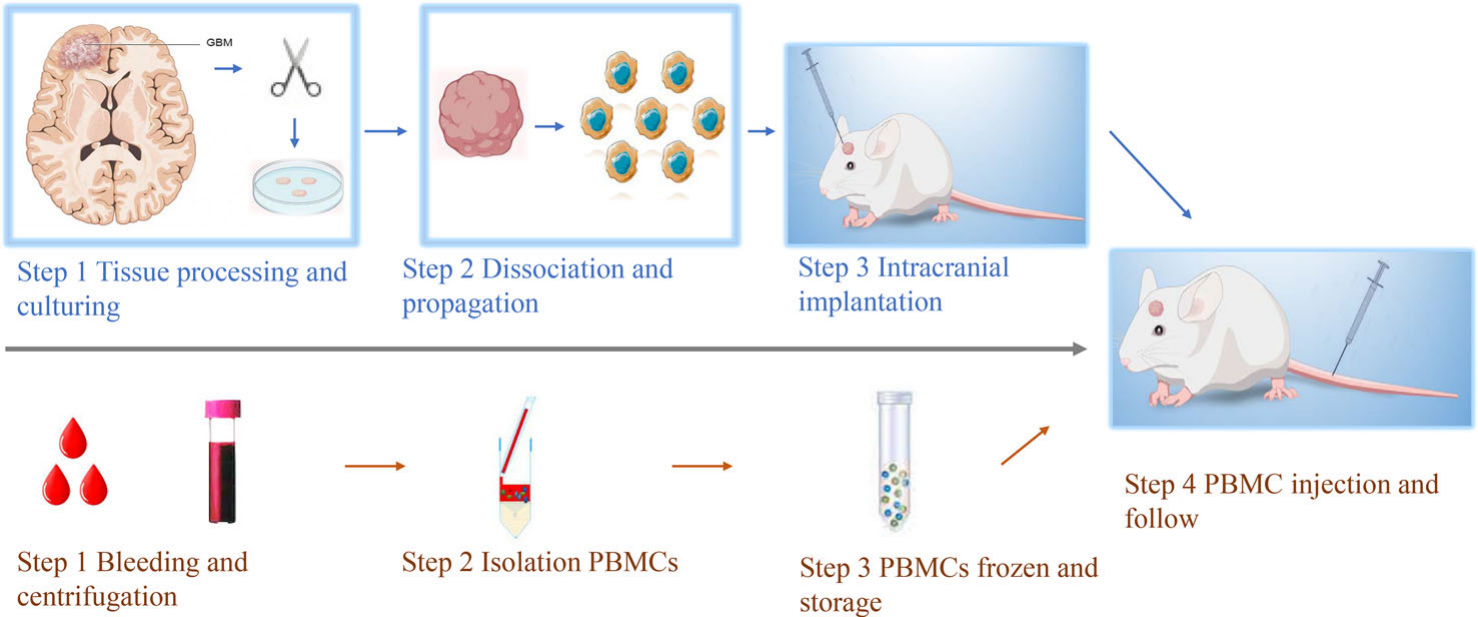
Workflow of establishing GBM HPDOX mice models. Top, the workflow of establishing the GBM PDOX model. Tumor specimens obtained from surgery were dissociated to be single tumor cells. Then tumor cells were cultured with proper conditions to enhance the formation of GBM neurospheres (Top step 1). After the obtainment of neurospheres could be tested (Top, steps 1 & 2). GBM neurospheres could be used to establish the PDOX model to study tumor biology or test novel medicine and instruments (Top, step 3). Bottom, the workflow of establishing patients’ PBMCs-derived humanized mouse models. The peripheral blood of GBM patients was collected and centrifugated (Bottom, step 1). The PBMCs were isolated and stored (Bottom, steps 2 & 3). After the establishment of the PDOX mice model for two weeks, the same patient’s PBMCs were injected into NSG mice for further study (step 4).

Establishment of GBM PDOX from patient’s tissues. The establishment of the PDOX model was conducted by injecting patients’ tumor-derived GBM cells into female immune-deficient nude mice. Candidate mice for PDOX models could be NSG, NOD-SCID, and nude. Herein we investigated *in vitro* culture of 26 PDOX generated and/or passaged in NSG mice. The workflow was shown in **Figure 1**, and details were described above. The demographic information of patients from whom PDOX was generated was summarized in **Table 1**. The age of patients ranged from 22 to 76 years old with a mean of 58 ± 12.05 years old, and 11 patients were women and 15 were men. The tumor volume varied from 4.88 to 115.33 cm^3^, with a mean of 48 ± 26.60 cm^3^. GBM cells from each specimen were injected into five mice, in which 12 specimens successfully inherited in mice. The overall engraftment rate of GBM PDOX in NSG mice was 46.15% (60/130). The total time of PDOX establishment ranged from 44 to 126 days, with a mean of 62 ± 17.5 days. Humanized GBM PDOX by transplanting the same patient’s PBMCs. Firstly, we established GBM PDOX mice. Two weeks later, the HPDOX models were established by engrafting the same patient’s PBMCs (**Figure 1**, bottom).

**Table 1:** The demographic and clinical information for patients of PDOX models.

Case ID	Age	Sex	Race	Pathology	Grades	Tumor	Smoke	Pretreatment	Passage	*In vivo*
						Volume (cm^3^)	status			Days
1	62	Male	Han	GBM	IV	11.76	Current	NO	–	–
2	70	Male	Tujia	GBM	IV	27.95	Former	NO	–	–
3	61	Female	Han	GBM	IV	31.4	Current	NO	F1	53
4	73	Male	Han	GBM	IV	115.33	Former	NO	F1	88
5	46	Female	Han	GBM	IV	15.17	Current	NO	F1	67
6	52	Male	Han	GBM	IV	100.68	Current	NO	F1	48
7	39	Female	Zhuang	GBM	IV	4.88	Never	NO	F1	44
8	22	Male	Han	GBM	IV	42.47	Never	Surgery/RT/CT	F1	56
9	64	Male	Han	GBM	IV	78.44	Current	NO	F1	68
10	67	Male	Han	GBM	IV	59.59	Current	NO	–	–
11	63	Female	Han	GBM	IV	29.97	Former	NO	F1	67
12	58	Male	Han	GBM	IV	47.38	Current	NO	F1	46
13	55	Female	Han	GBM	IV	47.36	Former	NO	F1	126
14	57	Female	Han	GBM	IV	56.76	Never	NO	F1	57
15	56	Male	Han	GBM	IV	62.2	Former	NO	F1	59
16	76	Female	Han	GBM	IV	105.13	Never	NO	F1	48
17	54	Male	Han	GBM	IV	29.47	Former	NO	F1	76
18	67	Female	Han	GBM	IV	33.43	Former	NO	F1	68
19	72	Male	Han	GBM	IV	41.79	Former	NO	–	–
20	73	Female	Han	GBM	IV	40.71	Current	NO	F1	67
21	64	Male	Tujia	GBM	IV	47.47	Former	NO	–	53
22	68	Female	Han	GBM	IV	18.54	Former	NO	F1	57
23	61	Male	Han	GBM	IV	35.87	Former	NO	F1	51
24	57	Female	Miao	GBM	IV	40.22	Current	NO	F1	49
25	43	Male	Han	GBM	IV	61.94	Former	NO	–	–
26	40	Male	Han	GBM	IV	49.39	Current	NO	F1	52

GBM, glioblastoma; RT, radiotherapy; CT, chemotherapy.

### Patients’ T Cells Reconstituted Well in GBM HPDOX Mice

**Figure 2:**
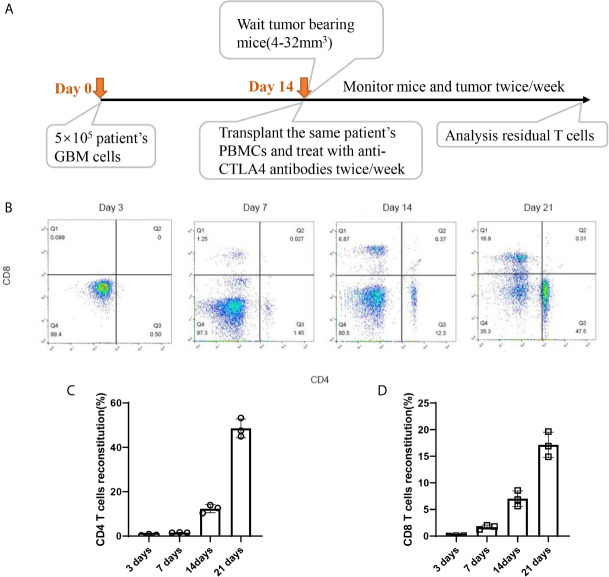
The reconstitution of T cells derived from GBM patients in HPDOX mice model. **(A)** Timepoint of the construction of HPDOX mice model. **(B)** Flow cytometry analysis of CD4^+^ and CD8^+^ T cells in NSG mice with transplanted PBMCs. **(C, D)** The population of CD4^+^ **(C)** and CD8^+^ **(D)** T cells in NSG mice with transplanted PBMCs. Each experiment was repeated at least three times, and three repeats were included each time.

Timepoint of the construction of HPDOX mice model. PBMCs were collected from GBM patients and the HPDOX model was constructed in PDOX mice bearing tumors derived from the same patients (**Figure 2A**). Flow cytometry of CD4+ and CD8+ T cells in HPDOX to explore the differentiated T cells within transplanted PBMCs (**Figure 2B**). The population of CD4+ (**Figure 2C**) and CD8+ (**Figure 2D**) T cells in GBM HPDOX mice. Our results showed both CD4+ and CD8+ T cells reconstituted well in GBM HPDOX mice.

### Evaluation of Antitumor Effects of Anti-CTLA4 Antibodies in HPDOX Mice Model

**Figure 3:**
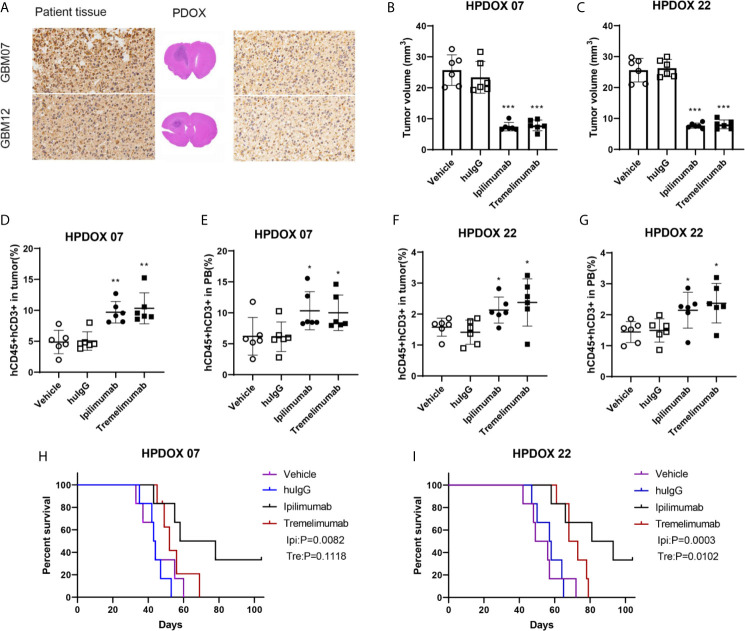
Evaluating the antitumor effect of anti-CTLA4 monoclonal antibodies in HPDOX. **(A)** IHC analyzes anti-CTLA4 in HPDOX 07, HPDOX 22; Scale bar: 20 μm (sides)/1,000 μm (middle). **(B, C)** Tumor volume of GBM xenograft in mice treated with Vehicle, huIgG, ipilimumab, and tremelimumab. **(D–G)** The population of T cells in GBM tissues **(D, F)** and peripheral blood **(E, G)** in mice treated with Vehicle, huIgG, ipilimumab, and tremelimumab. **(H, I)** The survival data of the HPDOX 07, and HPDOX 22 was presented after the treatment respectively. There were six mice in each group. Data were represented as mean ± SD. *P < 0.05, **P < 0.01, ***P < 0.001.

GBM07 and GBM22 were selected as the representation since they had a high expression of CTLA4 (**Table S1** and **Figure 3A**). After the construction of the HPDOX model, mice were treated with anti-CTLA4 antibody ipilimumab and tremelimumab twice weekly as the workflow described before. Results showed that tumor volume was significantly reduced after the application of ipilimumab and tremelimumab (*P <*0.05) (**Figures 3B, C**). After the intervention, residual human T cells (CD45+, CD3+) could be detected and their levels were significantly elevated in the tumor microenvironment and PB in HPDOX mice (*P <*0.05) (**Figures 3D–G**). Then we explored the population of the subgroups of the T cells. The activated T cells were presented with a higher expression of IFN-γ+ and CD25+. The population of the activated T cells was increased significantly after the treatment of anti-CTLA4 antibodies (*P <*0.01, **Figures S2A–C**). The exhausted T cells presented the lower expression of IFN-γ+ (**Figure S2B**). The exhausted T could differentiate into the activated T cells in certain conditions. The effector T cells are usually CD45RA positive, which was increased dramatically after treatment (*P <*0.001, **Figures S2A, D**). The memory T cells are CD45RO positive, which showed no significant difference between the groups (**Figures S2A, E**). The P value of the survival data of the HPDOX 07, and HPDOX 22 was 0.0082 and 0.0003 respectively, which indicated the significant difference between the treatment groups (**Figures 3H, I**). However, for the tremelimumab, the P value was 0.1118 and 0.0102 respectively, which indicated the significant difference between the treatment groups only in HPDOX 22 instead of HPDOX 07. These results indicated that CTLA4 played an inhibitory role in immune surveillance and HPDOX models could be appropriate approaches for revealing antitumor mechanisms and effects of anti-CTLA4 immunotherapy.

### FOXP3^+^ and Ratio of CD4^+^/CD8^+^ T Cells Were Associated With IRAEs

**Figure 4:**
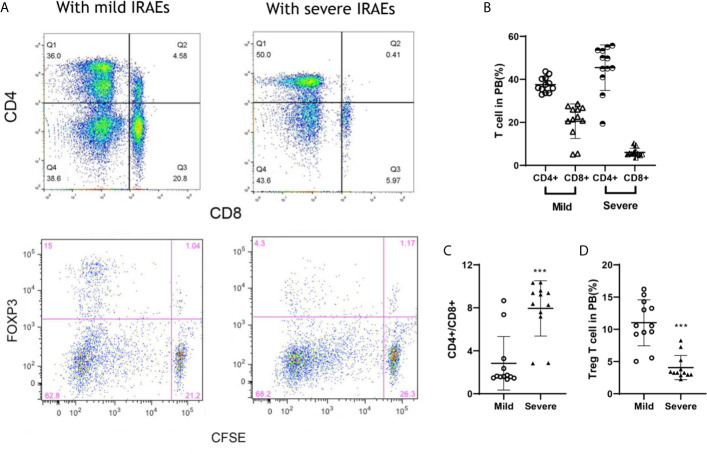
FOXP3+ and CD4+/CD8+ T cells were associated with IRAEs during anti-CTLA4 immunotherapy. **(A)** The population of CD4+, CD8+, and FOXP3+ T cells after the intervention of ipilimumab. **(B, C)** The population of T cells **(B)** and Treg cells **(C)** in peripheral blood of mice with IRAEs or not. **(D)** The ratio of CD4+/CD8+ T cells in peripheral blood of the HPDOX mice with IRAEs and not. There were twelve mice in each group. Data were represented as mean ± SD. ***P < 0.001.

To prolong the period for evaluation of the efficacy and IRAEs of immunotherapy, the detail of PBMCs transplantation is essential for the success of HPDOX (**Table 2**). The period can be prolonged to 47.90 ± 17.91 days engrafted with the decreased number (1 × 10^6^ vs >1 × 10^7^ normally) of autologous patients’ PBMCs (**Figure S3** and **Table 1**). Further, we detected the effect of ipilimumab at different doses in HPDOX mice models. The construction of HPDOX was detailed as before and GBM26 was taken as an example. Ten mice in each group were administrated with a high dose (10 μg/g) and normal dose (3 μg/g) of ipilimumab respectively. IRAEs were detected in 7/10 in the high dose group, only 1/10 in the normal dose group within the next two months (**Table 3**). After the intervention, we found that CD4+ and CD8+T cells reconstituted well in GBM HPDOX26 mice (**Figures 4A** top and **B**), and the ratio of CD4+/CD8+ T cells was dramatically increased in mice with IRAEs (*P <*0.05) (**Figure 4C**). For the mice without IRAEs, the CD4/CD8 ratio after therapy was 2.34 ± 2.12, which is the normal range of human. But for the mice with IRAEs, the ratio shifts to 8.06 ± 2.69. The CD4/CD8 ratio increased because the percentage of CD4 T cells increased, while the percentage of CD4 T cells decreased in PB. Moreover, the FOXP3+ Treg cells were decreased in mice with IRAEs (*P <*0.05) (**Figures 4A** bottom and **D**). These results indicated that the promotion of CD4+ T cells and the suppression of CD8+ and FOXP3+ Treg cells were associated with the occurrence of IRAEs. The construction of the HPDOX mice model could provide a platform for assessing adverse events of immunotherapies.

**Table 2:** Establishing autologous GBM HPDOX.

	Materials	Characteristics and delivery routes in the immunodeficient mouse	Analyses performed for HPDOX
Immunity	PBMCs by intraperitoneal	Human cells or cytokines by intravenous	Mature and function T cells and Treg Graft-versus-host disease
GBM	Patient derived orthotopic xenograft (PDOX)	Preclinical study of drugs and cells by intravenous	Weight loss Tumor growth Mutation Access therapeutic drugs
TME	PDOX +Autologous PBMCs by intraperitoneal	Preclinical study of drugs, cells, ICIs, and Vaccination by intravenous	Weight/hair loss Tumor growth Mutation Access therapeutic drugs Immune response IRAEs

TME, tumor microenvironments; ICIs, immune checkpoint inhibitors; IRAEs, immune-related adverse events.

**Table 3:** Evaluating the IRAEs under the treatment of ipilimumab.

IRAEs	Mild	Serve	Total	*P*-value
Low dose	10	2	12	0.0306
High dose	2	10	12	
Total	12	12	24	

IRAEs, immune-related adverse events.

### The Degree of IRAEs in HPDOX May Be Related to CD56^+^ Cells

**Figure 5:**
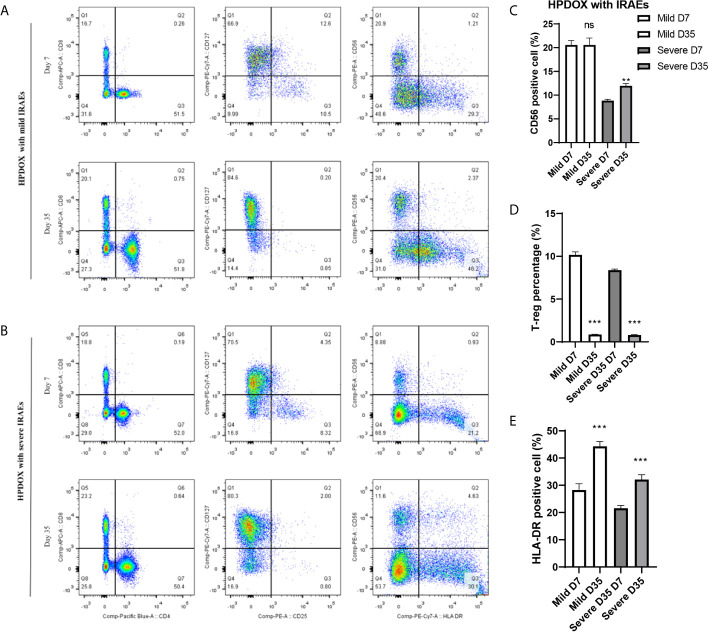
The degree of IRAEs may be related to CD56+ expression in HPDOX. **(A, B)** The population of CD4+, CD8+, the CD25+CD127− Treg cells, CD56, and HLA-DR in both mild and severe IRAEs groups after the intervention of ipilimumab. **(C)** There was no significant difference in the CD56 expression in the mild IRAEs group, although increased in the severe IRAEs group. **(D, E)** The CD25+CD127− Treg cells were significantly decreased, while the HLA-DR expression was increased in both mild and severe IRAEs groups. There were six mice in each group. Data were represented as mean ± SD. **P < 0.01, ***P < 0.001.

After the treatment of ipilimumab, the HPDOX with mild or severe IRAEs were accessed by flow cytometry to detect the possible mechanism of IRAEs. The whole blood was collected at the 7th and 35th days after the autologous PBMCs transplantation. As is shown in **Figure 5**, HPDOX suffered systemic IRAEs were assessed by the criterion of mouse GVHD clinical scoring system including weight loss, posture, activity, fur texture, and skin integrity (14). The CD4, CD8, CD25, CD127, CD56, and HLA-DR were detected to evaluate the expression of CD4, CD8 in T cells, the CD25+ CD127- Treg cells, CD56, and HLA-DR (**Figures 5A, B**). Those results showed, after the treatment of ipilimumab, there was no significant difference in the CD56 expression in the mild IRAEs group (**Figure 5C**), although increased in the severe IRAEs group. It indicates that the degree of IRAEs in HPDOX may be related to CD56+ cells. Similarly, the CD25+CD127- Treg cells were significantly decreased, while the HLA-DR expression was increased in both mild and severe IRAEs groups (**Figures 5D, E**).

## Discussion

During the past decades, various animal models have been developed to study brain tumor (15). Although established cell lines such as U87 and U251 have inherited most of the genetic and genomic features of GBM, they bear nothing in common with the actual patients’ GBM such as invasion histologic hallmarks (6). the development of severely immunocompromised mice has laid a solid foundation of PDOX, which has attracted more and more attention to facilitating biologic studies, preclinical drug evaluation, and biomarker identification (4–8). There are two main methods we have tried to obtain single cells for establishing the PDOX mice model: firstly, GBM tumor tissue right after they obtain from surgery is dissociated into single cells by treatment with Accutase™ solution to digest the extracellular material; secondly, the primary GBM cultures of tissue-derived cells which may acquire purer GBM cells but the resemblance disappeared with passages increasingly (6). The primary GBM culture conditions are also be used for the culture of GBM stem cells, which can enhance the success of produce those phenotypes from patients’ tissue in PDOX *via* few cells with enlarging tumor-initiating potential (4). Therefore, PDOX can reproduce biological features of GBM, such as brain invasion, microvascular proliferation, and anti-tumor therapy. Moreover, PDOX can simulate an appropriate microenvironment for cancer research compared with heterotopic extracranial implantation (16, 17). However, a limitation of PDOX should be addressed that it cannot be used in the researches on the immune of the microenvironment because of the requirement for immune-deficient nude mice as hosts (18).

Therefore, the HPDOX mice model was developed, which was a promising approach to facilitate the understanding of human immunity and the evaluation of the efficacy and IRAEs of immunotherapy *in vivo*. Stem-like cells derived HPDOX was reported with a low occurrence of IRAEs such as GVHD but represent few features of cancer in patients (19). HPDOX derived from patients’ PBMCs is the easiest way, which has limited application in following antigenic immune responses, but still is applied to access human immunosuppressive reagents (19). Although the novel mouse strains have been developed to inhibit the IRAEs such as GVHD by MHC complex-deficient mouse (20, 21), the immune cell differentiation and response could also be impeded. In this study, we established HPDOX mice models and evaluated the potential mechanism of anti-CTLA4 immunotherapy in GBM. Patients’ PBMCs could be engrafted into PDOX to reconstitute T cells for humanizing. Usually, the GVHD occurs 2–4 weeks after PBMC transfer. To prolong the period for evaluation of the efficacy and IRAEs of immunotherapy, the detail of PBMCs transplantation is essential for the success of HPDOX. The period can be prolonged after engrafted with the decreased number of autologous patients’ PBMCs. Specifically, IRAEs were assessed by weight loss, posture, activity, fur texture, and skin integrity. Although PBMCs derived humanized mice have been thoughted to be an appropriate platform to evaluate the efficacy of targeted therapy or immunotherapy, no previous literature has described its application in assessing the efficacy and IRAEs of immunotherapies in the GBM HPDOX mice model. The reconstitution of PBMCs to T cells is faster than stem-like cells, and it has higher veracity as a model (22). Similarly, our study revealed that the application of PBMCs could efficiently construct the HPDOX mice model to investigate the antitumor activities and IRAEs of anti-CTLA4 antibodies.

Immune checkpoint inhibitors (ICIs) enhance the anti-tumor immune response by blocking Treg-mediated immunosuppression. Why the T cells in the tumor microenvironment are few and non-sensitive still unrevealed. Recently, HPDOX has become a brand-new tool to assess cancer immunotherapy, which sets a robust foundation for cancer immune-related researches (23). In our study, we aim to demonstrate that HPDOX mice are appropriate to investigate the efficacy and IRAEs of anti-CTLA4 antibody therapies against GBM. Thus, this model can be used as a platform to evaluate whether patients benefit from certain targeted immunotherapy or not, which may provide a solid basis for clinical decisions. As one of the most common ICIs, anti-CTLA4 antibodies such as ipilimumab and tremelimumab have enhanced the anti-tumor immune response in both preclinical and clinical research and achieved unprecedented success. The achievement of ICIs mainly on account of two essential factors: 1) attenuate highly immunosuppressive tumor microenvironment by lowering frequencies of Treg cells; and 2) T effective cells are activated in tumor microenvironments (TME) by certain mechanisms, which play a critical role in anti-tumor in cellular immunity (24). Base on those points, anti-CTLA-4 antibodies might weaken their immunosuppressive effects *via* inhibiting the activated Treg cells.

Anti-CTLA4 antibodies block the CTLA4 molecules enhancing the anti-tumor immune response *via* inhibiting Treg-mediated immunosuppression in HPDOX. We observed the CTLA4 was blockaded by anti-CTLA4 antibodies and rescued the T cell exhaustion phenotype in the GBM HPDOX mouse model. Persistent exposure to high levels of antigen such as cancer or chronic infections may drive functional exhaustion of T cells (25). Recently, more and more researches focused on reversing T cell depletion abrogates the control of the proliferation of cancer. The critical role of CTLA4 in T cell exhaustion has been reported (26, 27). These findings warrant the clinical trial of CTLA4-targeted immunotherapy for GBM patients (NCT04606316). However, some patients do not benefit from anti-CTLA4 immunotherapy, which might because only a few T cells arrived in TME, which is not enough to reverse the immunosuppressive effects (28). In our study, ipilimumab, an anti-CTLA4 antibody that binds to CTLA4 specifically, showed the anti-GBM efficacy in inhibiting tumor growth *via* at least partly preserving T cells in the HPDOX model.

IRAEs showed a negative relationship with Treg numbers or percentages in the HPDOX model. This model may provide an emerging and promising tool to reveal the mechanism of clinical efficacy as well as IRAEs of immune-related therapies. IRAEs occurred in more than 90% of patients during the treatment with anti-CTLA4 antibody (29). Systemic administration of ICIs is usually not only influence by T cells in TME but also all T cells across the body. ICIs administrated by vein could induce IRAEs *via* unbalancing the T effect and Treg cells in normal tissues such as guts and skin. Those possible reasons reported for IRAEs during ICIs. On one side, Treg, expression of CTLA4, was inhibited by ADCC, which mediated by FcR expressing cells such as natural killer cells or macrophages in TME. On the other, T effective cells were activated and sustained *via* blocking the CTLA4 pathway. IRAEs could be induced by losing the functions or numbers of Treg, which are critical for maintaining tolerance (30). Based on those points, the dual roles of Treg are presented. On one hand, Treg cells can impede anti-tumor immunity to enhance immune evasion of tumor cells; On the other, Treg cells sustain an immune tolerance state and prevent from IRAEs. It reported that a negative relationship between Treg and IRAEs has been demonstrated in preclinical models, but not in GBM. Our study showed the patients’ PBMCs can co-exist in GBM HPDOX mice, which may be a useful platform for investigating the mechanism and role of the immune-related factors in IRAEs. In theory, it is a promising area to reform anti-CTLA4 antibodies and enhance its efficacy of cancer immunotherapy. To weaken ADCC and CDC effects, the N298A (Human IgG control) mutation was designed to prevent immune cells from IRAEs. However, our study showed N298A mutation did not affect the IRAEs of anti-CTLA4 antibodies, which might because patients’ natural killer cells or complement were not well restored in the HPDOX mice model (**Figure S4** and **Table S2**). And the degree of IRAEs may be related to CD56+ expression in HPDOX.

The challenge of immunotherapy is to prevent IRAEs while preserving anti-tumor efficacy. In theory, the immunosuppressors or corticosteroids probably maintain immune tolerance in normal organs and tissues, which could also impede the antitumor efficacy. But they are not weakening the antitumor efficacy of ICIs. Interestingly, some clinical trials showed a positive relationship between the IRAEs and antitumor responses (31, 32). It’s a promising direction to study this mechanism in the future (33, 34). In the future, our study will also aim to make the current anti-CTLA4 immunotherapy more effective *via* inhibiting Treg cells and weaken their immunosuppressive effects. In the meanwhile, activating T effective cells by ICIs or vaccine in TME.

However, some important limitations should be considered. In the first place, in the tissues and plasma of mice, there are only low levels of human factors and cytokines resulted in a decreased number of myeloid cells and Treg cells. With the presence of GM-CSF, IL-6, IL-3, and M-CSF, the differentiation of T, B, and NK cells were more actively (35, 36). Secondary, although the novel mouse strains have been developed to inhibit the GVHD by mouse MHC complex, the immune cell types and responses in HPDOX still need to be further studied (12, 37). Thirdly, accessing the change in the transcriptome and epigenome of the patient tumor tissues is passaged in the HPDOX models (19, 38). Lastly, personalized medicine strategies will be needed to allow higher tumor infiltration and anti-tumor responses.

## Conclusions

In conclusion, our study established HPDOX mice models for investigating the mechanism and IRAEs of immunotherapies in GBM, which would offer a promising platform for evaluating the efficacy and IRAEs of novel therapies and exploring personalized therapeutic strategies.

## Data Availability Statement

The original contributions presented in the study are included in the article/**Supplementary Material**. Further inquiries can be directed to the corresponding author.

## Ethics Statement

The studies involving human participants were reviewed and approved by the Ethics Committee of Xiangya Hospital. The patients/participants provided their written informed consent to participate in this study. The animal study was reviewed and approved by the Ethics Committee of Xiangya Hospital.

## Author Contributions

CL conceived, designed, and supervised the study. SX and XY drafted the manuscript. SX and XY performed data analysis. SX, XY, and GD collected the data. All authors contributed to the article and approved the submitted version.

## Funding

This work was supported by the National Natural Science Foundation of China (81902553); Natural Science Foundation of Hunan Province (2019JJ50942).

## Conflict of Interest

The authors declare that the research was conducted in the absence of any commercial or financial relationships that could be construed as a potential conflict of interest.

## Supplementary Material

Supplementary Figure 1 The tumor volume before antibodies treatment. **(A)** The tumor volume of PDOX 07; **(B)** The tumor volume of PDOX 22. There were three mice in each group. Data were represented as mean ± SD. ns: *P* > 0.05.

Supplementary Figure 2 The subgroups of T cells. **(A–C)** The activated T cells were presented with the higher expression of IFN-γ+ and CD25+. **(B)** The exhausted T cells presented the lower expression of IFN-γ+. (a & d) The effector T cells are CD45RA positive. (a & e) The memory T cells are CD45RO positive.

Supplementary Figure 3 HPDOX with IRAEs such as graft-versus-host disease (GVHD). (left) The normal autologous GBM HPDOX without IRAES like GVHD. (right) The autologous GBM HPDOX mice suffered IRAES and/or GVHD. Specifically, IRAEs was assessed by weight and hair loss, posture, activity, fur texture and skin integrity.

Supplementary Figure 4 Evaluating the antitumor effect of ipilimumab without and with the N298A mutation in HPDOX. **(A, B)** Tumor volume of GBM xenograft in mice treated with Vehicle, huIgG, ipilimumab, and ipilimumab with N298A. **(C, E)** The population of T cells in GBM tissues and **(D, F)** peripheral blood in mice treated with Vehicle, huIgG, ipilimumab, and ipilimumab with N298A. There were six mice in each group. Data were represented as mean ± SD. ns, no significant difference.

## Abbreviations

PDOX, patient-derived orthotopic xenograft; GBM, glioblastoma; HPDOX, humanized patient-derived orthotopic xenograft; Treg, regulatory T; IRAEs, immune-related adverse events; PBMCs, peripheral blood mononuclear cells; PB, peripheral blood; GVHD, graft-versus-host disease; ICI, immune checkpoint inhibitors.
