# Supplementary figures and images for: A Novel Mice Model for Studying the Efficacy and IRAEs of Anti-CTLA4 Targeted Immunotherapy
Source: Front Oncol. 2021 Jun 10;11:692403. doi: 10.3389/fonc.2021.692403 (PMC8222697; doi:10.3389/fonc.2021.692403)

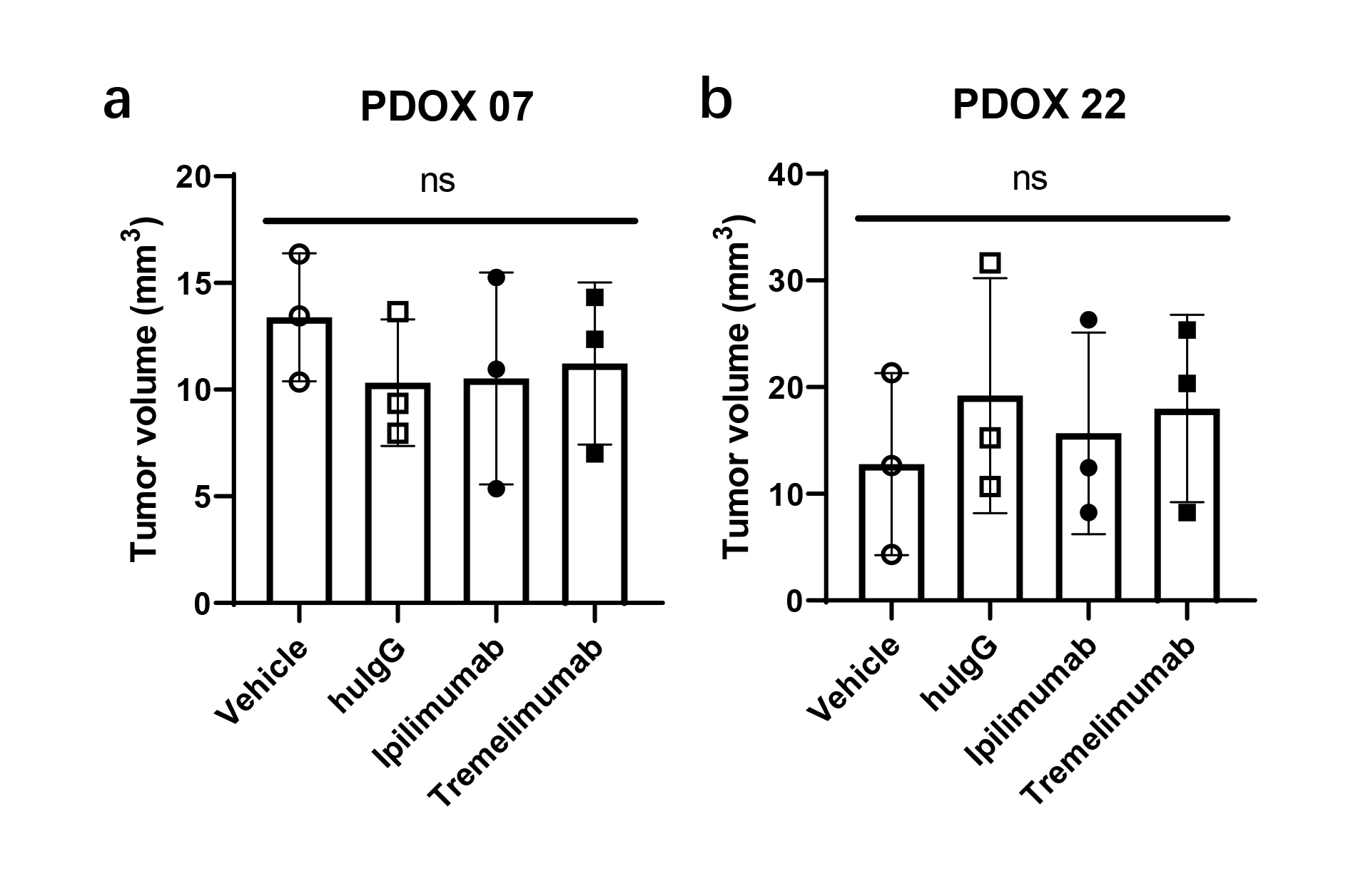

Supplement: Supplementary Figure 1 — The tumor volume before antibodies treatment. (A) The tumor volume of PDOX 07; (B) The tumor volume of PDOX 22. There were three mice in each group. Data were represented as mean ± SD. ns: P > 0.05. [file Image_1.tif]

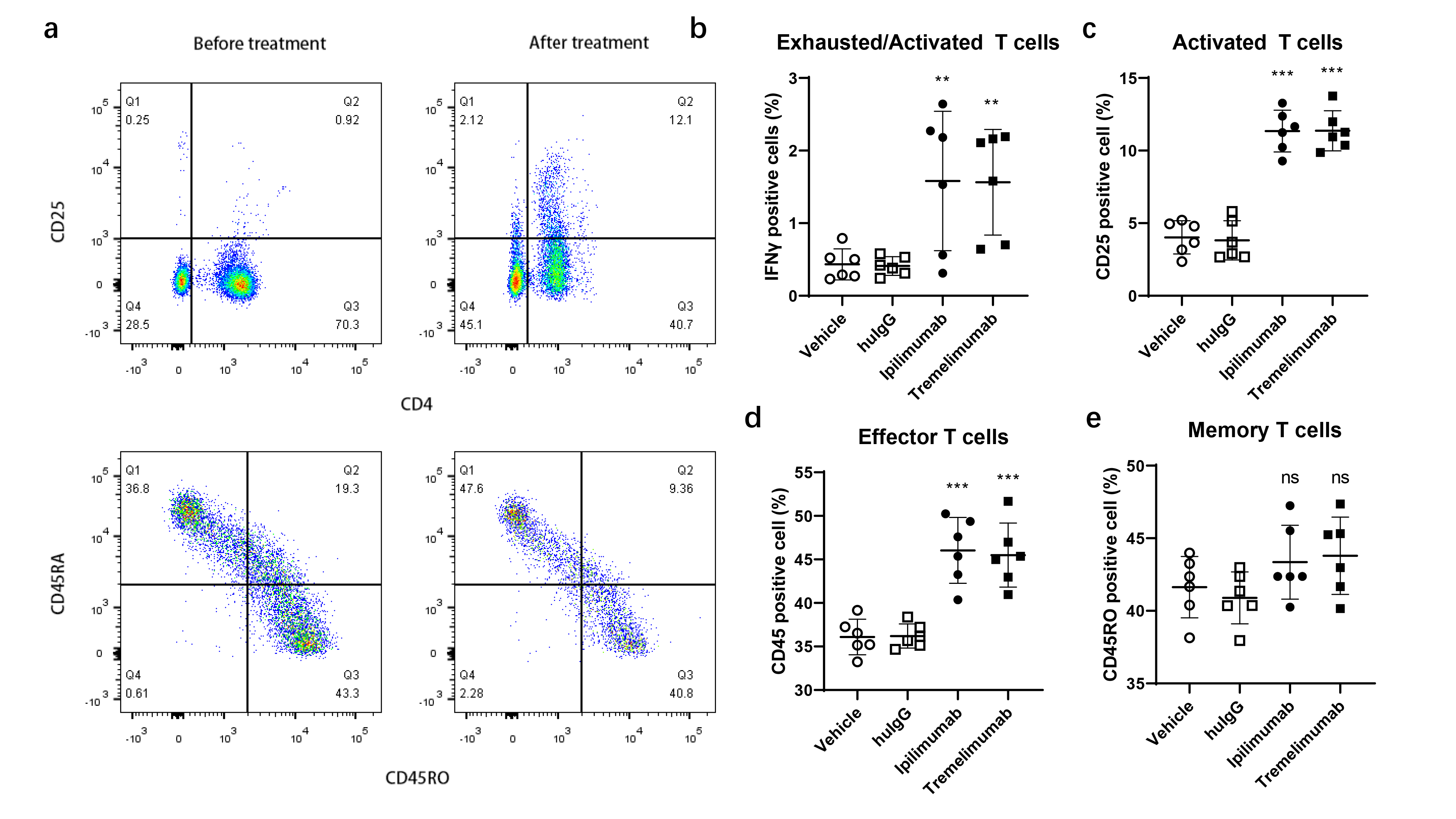

Supplement: Supplementary Figure 2 — The subgroups of T cells. (A–C) The activated T cells were presented with the higher expression of IFN-γ+ and CD25+. (B) The exhausted T cells presented the lower expression of IFN-γ+. (a & d) The effector T cells are CD45RA positive. (a & e) The memory T cells are CD45RO positive. [file Image_2.tif]

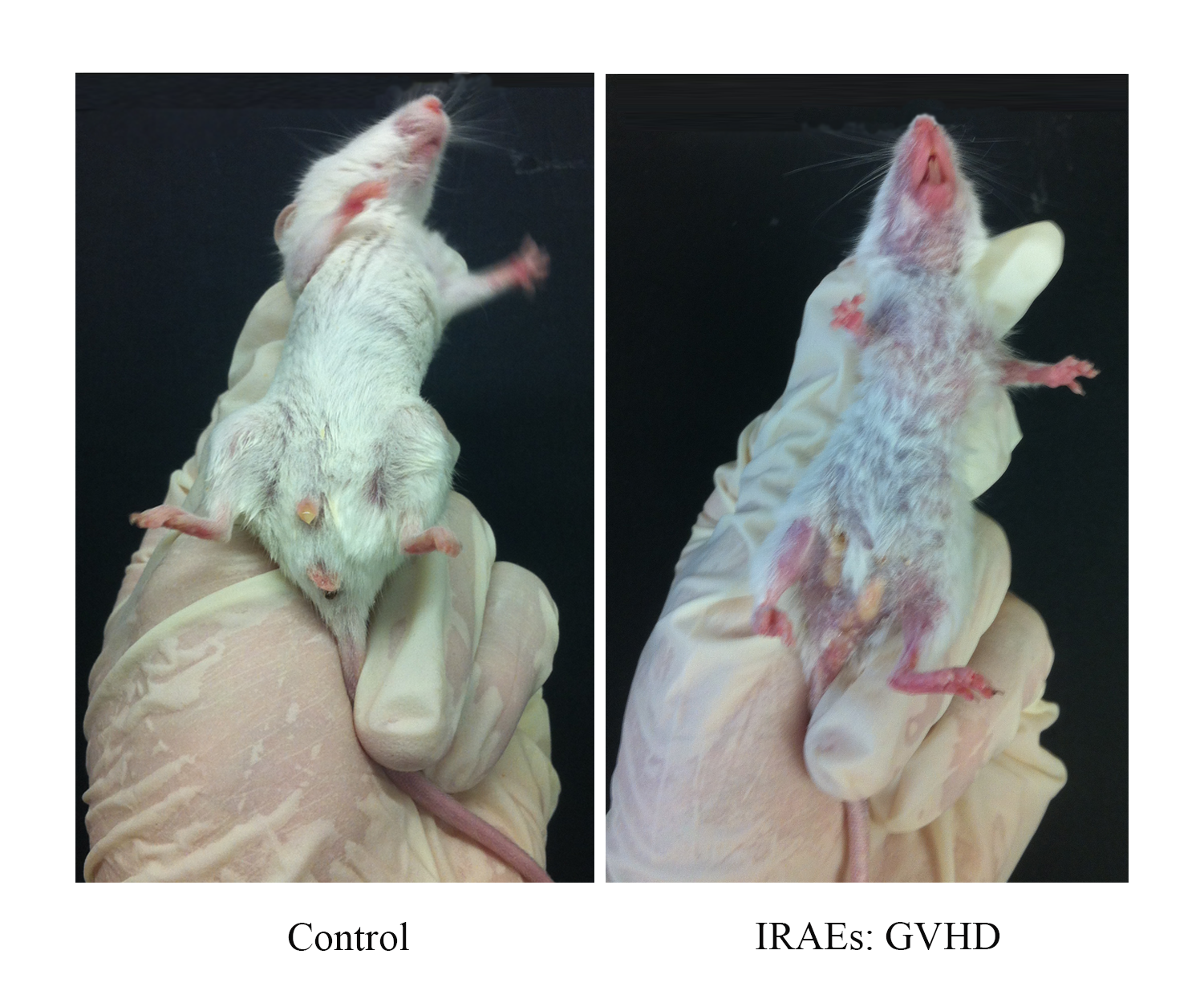

Supplement: Supplementary Figure 3 — HPDOX with IRAEs such as graft-versus-host disease (GVHD). (left) The normal autologous GBM HPDOX without IRAES like GVHD. (right) The autologous GBM HPDOX mice suffered IRAES and/or GVHD. Specifically, IRAEs was assessed by weight and hair loss, posture, activity, fur texture and skin integrity. [file Image_3.tif]

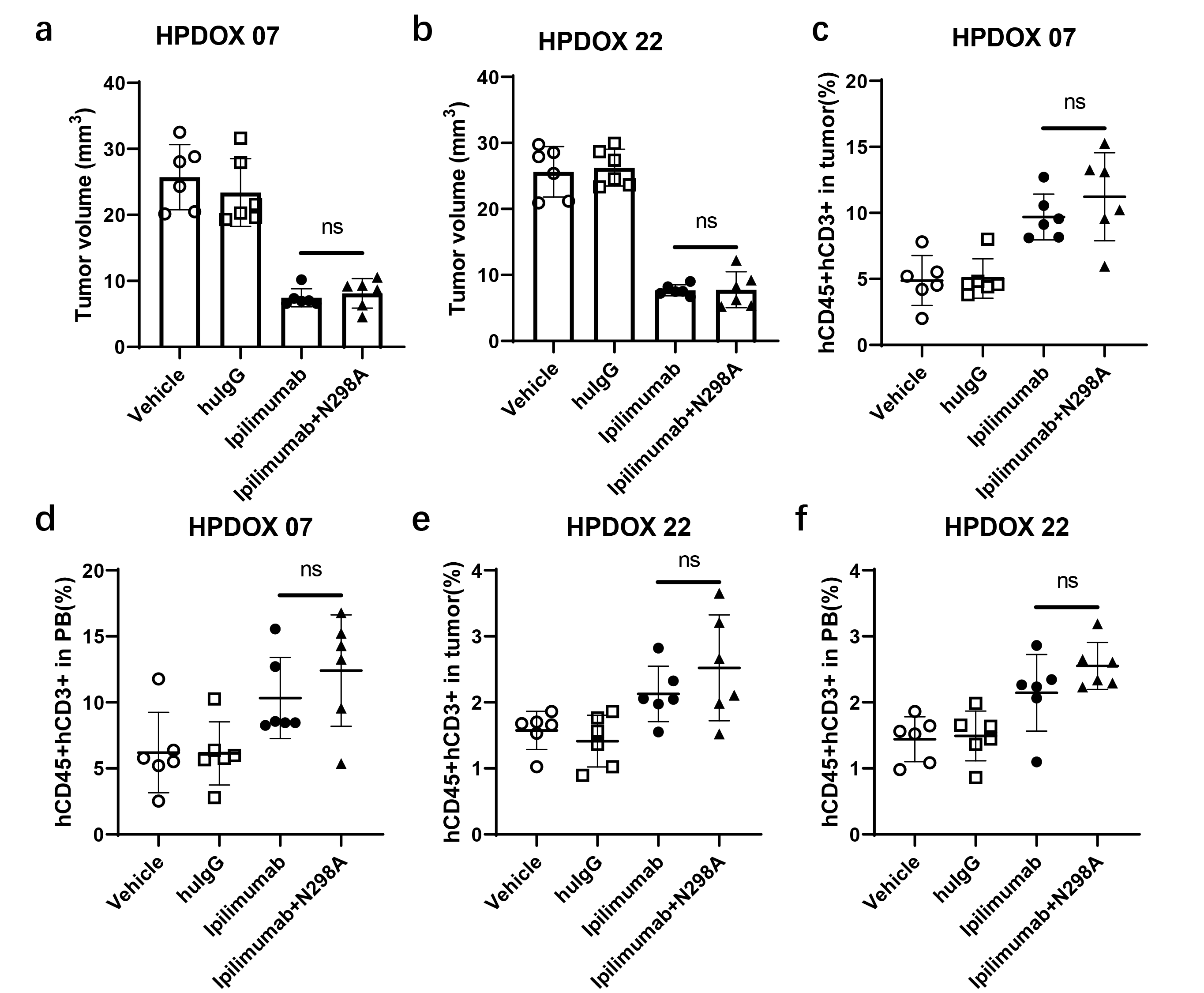

Supplement: Supplementary Figure 4 — Evaluating the antitumor effect of ipilimumab without and with the N298A mutation in HPDOX. (A, B) Tumor volume of GBM xenograft in mice treated with Vehicle, huIgG, ipilimumab, and ipilimumab with N298A. (C, E) The population of T cells in GBM tissues and (D, F) peripheral blood in mice treated with Vehicle, huIgG, ipilimumab, and ipilimumab with N298A. There were six mice in each group. Data were represented as mean ± SD. ns, no significant difference. [file Image_4.tif]
