# Supplementary material for: A Novel Mice Model for Studying the Efficacy and IRAEs of Anti-CTLA4 Targeted Immunotherapy
Source: Front Oncol. 2021 Jun 10;11:692403. doi: 10.3389/fonc.2021.692403 (PMC8222697; doi:10.3389/fonc.2021.692403)
Supplement: Supplementary file 5 [file Table_1.docx]

| Table S1. Information of patients whose samples were used to establish HPDOX model. | | | |
| --- | --- | --- | --- |
| Sample | Gender | Age | Histology |
| HPDOX 07 | Male | 39 | Glioblastoma |
| HPDOX 22 | Male | 68 | Glioblastoma |

| Table S2. Evaluating the IRAEs under the treatment of ipilimumab with or without N298A | | | | |
| --- | --- | --- | --- | --- |
| IRAEs | Mild | Severe | Total | p value |
| Ipilimumab | 10 | 2 | 12 | 0.9261 |
| Ipilimumab+N298A | 8 | 4 | 12 |  |
| Total | 17 | 7 | 24 |  |

IRAEs: immune-related adverse events
